# Impact of 2009 pandemic influenza among Vietnamese children based on a population-based prospective surveillance from 2007 to 2011

**DOI:** 10.1111/irv.12244

**Published:** 2014-03-07

**Authors:** Minh Nhat Le, Lay Myint Yoshida, Motoi Suzuki, Hien Anh Nguyen, Huu Tho Le, Hiroyuki Moriuchi, Duc Anh Dang, Koya Ariyoshi

**Affiliations:** a Institute of Tropical Medicine, Nagasaki UniversityNagasaki, Japan; b National Institutes of Hygiene and EpidemiologyHanoi, Vietnam; c Khanh Hoa Health Service DepartmentNha Trang, Vietnam; d Graduate School of Biomedical Sciences, Nagasaki UniversityNagasaki, Japan

**Keywords:** Influenza, pandemic, pediatric, Vietnam

## Abstract

**Background:**

Influenza virus is one of the major viral pathogens causing pediatric acute respiratory infection (ARI). The spread of pandemic influenza A (A(H1N1)pdm09) in 2009 around the globe had a huge impact on global health.

**Objective:**

To investigate the impact of A(H1N1)pdm09 on pediatric ARI in Vietnam.

**Study design:**

An ongoing population-based prospective surveillance in central Vietnam was used. All children aged <15 years residing in Nha Trang city, enrolled to the ARI surveillance in Khanh Hoa General Hospital, from February 2007 through March 2011 were studied. Clinical data and nasopharyngeal swab samples were collected. Influenza A was detected and genotyped by multiplex polymerase chain reaction assays and sequencing.

**Results:**

Among enrolled 2736 hospitalized ARI cases, 354 (13%) were positive for influenza A. Genotyping results revealed that seasonal H3N2 and H1N1 (sea-H1N1) viruses were cocirculating before A(H1N1)pdm09 appeared in July 2009. The A(H1N1)pdm09 replaced the sea-H1N1 after the pandemic. The majority of influenza A cases (90%) were aged <5 years with incidence rate of 537 (387–775) per 100 000 population. Annual incidence rates of hospitalized influenza cases for pre-, initial and post-pandemic periods among children aged <5 year were 474, 452, and 387 per 100 000, respectively. Children with A(H1N1)pdm09 were elder, visited the hospital earlier, less frequently had severe signs, and were less frequently associated with viral coinfection compared with seasonal influenza cases.

**Conclusions:**

The A(H1N1)pdm09 did not increase the influenza annual hospitalization incidence or disease severity compared with seasonal influenza among pediatric ARI cases in central Vietnam.

## Background

In April 2009, the pandemic influenza A (A(H1N1)pdm09) spreads rapidly across the globe.^1^,^2^ As of July 9, 2010, worldwide more than 214 countries have confirmed A(H1N1)pdm09 cases including over 18 311 deaths.^3^ However, there were notable differences in disease severity between in America and in Asia.^4^ In the United States, the number of deaths due to A(H1N1)pdm09 among children was nearly four times greater than the previous seasonal influenza seasons.^5^ The hospitalization and death rates for A(H1N1)pdm09 among Argentina children less than 1 year of age increased twice and 10 times, respectively, compared with the previous seasonal influenza.^6^ On the other hand, in Japan, even though large outbreaks of pandemic influenza caused a high rate of hospitalization especially among children aged 5–9 year (59%), a very low number of deaths were reported.^7^ Similarly, a study from South India also reported that A(H1N1)pdm09 was associated with a low risk of hospitalization and ICU admission.^8^ Influenza outbreak during the immediate post-pandemic period has been known to cause severe morbidity and mortality.^9^ However, systematic analysis showed that the impact of A(H1N1)pdm09 in the post-pandemic period also varied across regions. Most of countries reported a lower influenza activity^10^,^11^, and some countries such as Spain, England, and Taiwan reported a shift in age distribution and severity.^12^–^14^

There were several published studies evaluating the impact of A(H1N1)pdm09; however, only few of them were population-based multiyear surveillance.^15^,^16^ We have established a community-based, prospective surveillance for childhood acute respiratory infections (ARI) in Nha Trang city, Khanh Hoa province, central Vietnam since February 2007. Because the area was surrounded by the sea and mountains and there was only a single hospital providing primary, secondary, and tertiary care for residents, we were able to determine the incidence rates of hospitalized ARI.^17^ This ARI surveillance has observed the A(H1N1)pdm09 outbreak and the post-pandemic period.

## Objectives

We conducted this study to describe the clinical pictures of A(H1N1)pdm09-infected children in comparison with those of seasonal influenza cases and to evaluate the impact of the pandemic influenza on the hospitalized influenza incidence among Vietnamese children.

## Study design

### Study population

Our study site covers the 16 communes of Nha Trang City in Khanh Hoa Province, central Vietnam. According to our census survey in 2006, the total population in the catchment area was 198 729 with 48 260 aged <15 years and 13 935 aged less than 5 years.^17^ Temperature is high throughout the year (average 26°C), and rainy season is from September to December. May to August is hot and dry, and January to March is relatively cool. Influenza vaccine had not been included in the national immunization program and was rarely used in our setting at the time of study.

A prospective surveillance for pediatric ARIs was initiated at the Khanh Hoa General Hospital (KHGH) in February 2007. In Vietnam, commune health centers and polyclinics provide primary and outpatient managements, while severe cases are referred to district or provincial hospitals. The KHGH is the only hospital in the area providing tertiary care for residents. Details of the surveillance methods were described previously.^18^ Mild influenza cases with fever and sore throat were treated at outpatient department of KHGH and polyclinics in the community. Antibacterial agents could be prescribed but antivirals were not available. In brief, all hospitalized children who presented with cough and/or difficulty breathing, aged less than 15 year and residing in the catchment area, were enrolled. This covered all pediatric influenza cases that required hospitalization. Demographic and clinical information were collected by trained research nurses using a standardized form. Blood and nasopharyngeal swab (NSP) specimens were obtained from the participants after admission and completion of informed consent. Flexible Sterile FLOQ Swab, COPAN, Italy (#503CS01) was used for the nasopharyngeal sample collection. Chest X-rays were taken within 48 hours on admission and read in accordance with the WHO radiological reading guideline, radiologically confirmed pneumonia indicated end point consolidation.

### Laboratory testing

Collected NSP samples were processed within 3 hours and stored at −80°C. Nucleic acid extraction was performed using QIAamp viral RNA extraction kit (QIAGEN, Hilden, Germany). Four multiplex RT-PCR assays (I): influenza A (influ-A), influenza B virus (influ-B), respiratory syncytial virus (RSV), human metapneumovirus (hMPV); (II): parainfluenza viruses (PIV)-1, PIV-2, PIV-3, and PIV-4; (III): human rhinovirus (HRV), human coronavirus 229E (HCoV-229E), human coronavirus OC43 (HCoV-OC43); (IV): human adenovirus (hAdV) and human bocavirus (HBoV) were performed to detect influenza virus and 12 other respiratory viruses in each sample, as described previously.^18^ This assays targeted the influ-A matrix gene, so it can detect all influenza A viruses including A(H1N1)pdm09. Influ-A-positive samples were further genotyped by RT-PCR and sequencing of the influenza HA and NA genes using previously published methods.^19^ RNA extracted directly from the clinical samples was used for the genotyping.

### Data management and statistical analysis

All data were double entered and validated by trained data management staffs at the Khanh Hoa Provincial Health Service using foxpro 9.0 (Microsoft, Redmond, WA, USA) databases. Characteristics of patients were compared using chi-squared tests and *t*-tests. The census population was treated as a cohort for the calculation of incidence rates. The 95% confidence intervals for incidence rate were calculated using Wilson's score method. stata 10 (Stata corp., College Station, TX, USA) was used for all statistical analyses.

## Results

### Case enrollment and influenza virus testing

**Figure 1:**
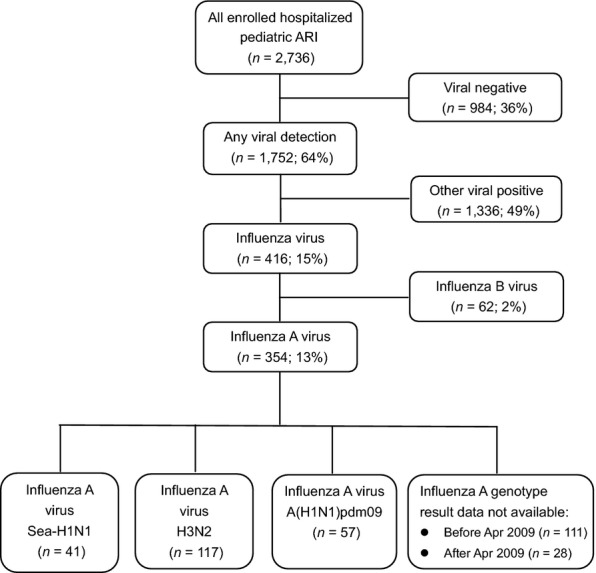
Flow diagram of enrolled hospitalized pediatric acute respiratory infection (ARI) cases at Khanh Hoa General Hospital during the study period.

During the study period, a total of 2736 hospitalized ARI cases were enrolled of which 354 (12·9%) were positive for influ-A. Influenza A genotyping was performed, and 215 (61%) of the samples were genotyped of which 57 (16%) were A(H1N1)pdm09, 117 (33%) were H3N2, and 41 (12%) were seasonal H1N1 (Figure 1). A total of 139 samples consisting of 111 before and 28 after pandemic influenza outbreak were not applicable to genotyping. The first case A(H1N1)pdm09 was detected in Vietnam on May 31, 2009.^20^ However, in this study, we used April 2009 as the timescale for the appearance of A(H1N1)pdm09 since the first case of A(H1N1)pdm09 was reported in April 2009 in Mexico. Therefore, 111 cases of influenza A before April 2009 that were not applicable for genotyping were regarded as seasonal influenza for clinical data analysis.

### Seasonality of influenza A viruses

**Figure 2:**
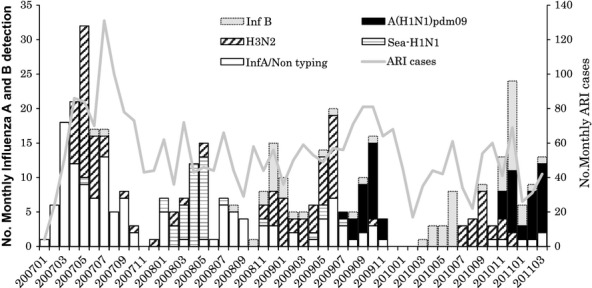
The patterns of seasonal H3N2 and H1N1, and A(H1N1)pdm09 influenza virus infections among hospitalized acute respiratory infection (ARI) cases at Khanh Hoa General Hospital from February 2007 through March 2011.

Before the 2009 pandemic influenza outbreak, seasonal H1N1 (sea-H1N1) and H3N2 were cocirculating in the region. Influenza seems to be present throughout the year, and its peak season is usually around May and also December in 2008 (Figure 2). The A(H1N1)pdm09 was detected first in July 2009, peaked in October 2009, and gradually faded in December 2009 in Nha Trang. In 2010, there was a wave of influenza B outbreak from March 2010 to May 2010. Then, H3N2 influenza A appeared in July 2010 to December 2010. The second wave of A(H1N1)pdm09 started in November 2010 and went up till March 2011.

### Incidence of influenza A among hospitalized pediatric ARI cases

The detailed annual incidence rates of hospitalized influenza A cases are shown in Table 1. The incidence was highest among children aged 12–23 months (1132 per 100 000) followed by those aged <11 months (857 per 100 000). This pattern was similar for both pandemic and seasonal influenza cases. To elucidate the impact of A(H1N1)pdm09 on pediatric ARI hospitalization, we defined the time periods into three periods as pre-pandemic – seasonal (April 2008 to March 2009), initial (from April 2009 to March 2010), and post-pandemic influenza (April 2010 to March 2011) seasons. Annual incidence of hospitalized influenza cases per 100 000 population estimated for each periods among children aged <5 year was 474, 452, and 387, respectively.

**Table 1:** Incidence of Influenza A virus infections among hospitalized pediatric ARI in different age groups

Age group	Population*	Incidence per 100 000 population (95% CI)									
		Overall February 2007 to March 2011		February 2007 to March 2008		April 2008 to March 2009		April 2009 to March 2010		April 2010 to March 2011	
Months	*n*	Cases	Incidence	Cases	Incidence	Cases	Incidence	Cases	Incidence	Cases	Incidence
≤11	2250	82	857 (693–1059)	40	1422 (1047–1929)	17	756 (472–1207)	12	533 (305–930)	13	578 (338–986)
12–23	2431	117	1132 (948–1350)	50	1645 (1251–2161)	21	864 (566–1317)	23	946 (631–1416)	23	946 (631–1416)
24–59	9254	119	303 (253–362)	45	389 (291–520)	28	303 (209–437)	28	303 (209–437)	18	195 (123–307)
<60	13 935	318	537 (482–598)	135	775 (655–916)	66	474 (372–602)	63	452 (353–578)	54	387 (297–505)
≥60	38 690	36	22 (16–30)	11	23 (13–41)	7	18 (9–37)	12	31 (18–54)	6	16 (7–34)
Total	48 260	354	173 (156–191)	146	242 (206–285)	73	151 (120–190)	75	155 (124–195)	60	124 (97–160)

ARI, acute respiratory infection.

* Data from population census survey in 2006.

An increased hospitalization incidence of influenza A was observed among over 5 years age group compared with 2009 pre-pandemic periods (18–23 versus 31/100 000). Similarly, during two years period after 2009 pandemic outbreak: from April 2009 to March 2011, the estimated incidence rates for seasonal and pandemic influenza viruses among hospitalized ARI children aged <5 year were similar: 161 and 165, respectively, but there was an increase in incidence among the children older than 5 years age group (7 versus 16/100 000) (Table 2).

**Table 2:** Incidence of pandemic and seasonal influenza A viruses among hospitalized pediatric ARI in different age groups during pandemic and post-pandemic periods

Age group	Population*	Incidence per 100 000 population (95% CI)											
		Overall				First year of outbreak				Second year of outbreak			
		April 2009 to March 2011				April 2009 to March 2010				April 2010 to March 2011			
		Seasonal		Pandemic		Seasonal		Pandemic		Seasonal		Pandemic	
Months	*n*	Cases	Incidence	Cases	Incidence	Cases	Incidence	Cases	Incidence	Cases	Incidence	Cases	Incidence
≤11	2250	9	200 (105–379)	9	200 (105–379)	5	222 (94–519)	2	89 (24–323)	4	118 (96–456)	7	311 (151–641)
12–23	2431	19	390 (250–609)	14	288 (172–682)	8	329 (167–648)	6	247 (113–537)	11	452 (253–808)	8	329 (167–648)
24–59	9254	17	92 (57–147)	23	124 (83–186)	11	119 (66–213)	11	119 (66–213)	6	56 (30–141)	12	130 (74–227)
<60	13 935	45	161 (120–216)	46	165 (124–220)	24	172 (116–256)	19	136 (87–213)	21	150 (99–230)	27	194 (133–282)
≥60	34 325	5	7 (3–17)	11	16 (9–28)	4	11 (5–30)	6	17 (8–38)	1	3 (0–15)	5	15 (6–34)
Total	48 260	50	52 (39–68)	57	59 (46–76)	28	58 (40–84)	25	52 (35–76)	22	46 (30–69)	32	66 (47–94)

ARI, acute respiratory infection.

* Data from population census survey in 2006.

### Demographic, clinical characteristics, and viral coinfection status

To determine the clinical severity of A(H1N1)pdm09 compared with seasonal influenza cases, we categorized the influenza A-positive samples into two groups as seasonal (H3N2 and H1N1, *n* = 269) and pandemic (A(H1N1)pdm09, *n* = 57) and performed the analysis (Table 3). The A(H1N1)pdm09 cases were older (40·8 versus 27·5 months, *P* = 0·0009), and the duration for onset to hospitalization was shorter (1·6 versus 3·1 days, *P* = 0·0015) compared with seasonal influenza A cases. However, the A(H1N1)pdm09 cases had lower body temperature (38·4 versus 39·7°C, *P* = 0·0003), slower pulse rate (98·6 versus 114·2/minute, *P* = <0·0001), and less wheeze (*P* = <0·0001) compared with seasonal influenza cases. The percentage of radiological confirmed pneumonia (RCP) cases among the A(H1N1)pdm09 was lower than the seasonal influenza viruses (11% versus 27%, *P* = 0·011). There was no difference in terms of hospitalization periods between the two groups. No influenza-positive ARI cases in this study were severe enough to received intensive care or required mechanical ventilation. Among a total of 354 influ-A cases, coinfection with other respiratory viruses was identified in 84 cases (24%). Respiratory syncytial virus was the commonest followed by HRV. Interestingly, coinfection with other viruses was seen less frequently among the A(H1N1)pdm09 cases compared with seasonal influenza cases (11% versus 31%, *P* = 0·001).

**Table 3:** Demographic and clinical characteristic of children <15 years infected with seasonal H3N2 and H1N1, and pandemic influenza (A(H1N1)pdm09)

Characteristic	Seasonal influenza (H3N2, H1N1)	Pandemic influenza A(H1N1)pdm09	*P*-value
	*n* = 269	*n* = 57	

	*n*/Mean (%/SD)		
Gender			
Male	165 (61)	33 (58)	0·629
Female	104 (39)	24 (42)	
Age (month)	27·5 (25·5)	40·8 (35·9)	**<0·001**
Duration for onset to admission	3·1 (0·3)	1·6 (2·3)	**0·001**
Clinical findings			
Body temperature (°C)	38·4 (0·8)	37·9 (0·9)	**<0·001**
Pulse rate (per minute)	114·2 (11·7)	98·6 (12·7)	**<0·001**
Wheeze			
Yes	145 (54)	9 (16)	**<0·001**
No	124 (46)	48 (84)	
WBC	11·7 (5·6)	10·3 (5·2)	0·089
RBC	4·5 (0·8)	4·5 (0·5)	0·917
Radiographically confirmed pneumonia (RCP)			
Yes	72 (27)	6 (11)	**0·011**
No	197 (73)	51 (89)	
Management			
Hospitalized period (days)	5·1 (3·0)	5·7 (8·0)	0·3
Number of household members	5·1 (2·0)	4·8 (2·7)	0·31
Breast feeding			
Yes	79 (30)	8 (14)	**0·017**
No	189 (70)	49 (86)	
Kindergarten attendance			
Yes	142 (53)	35 (62)	0·236
No	127 (47)	22 (38)	
Antibiotics given before admission			
Yes	103 (38)	19 (33)	0·482
No	166 (62)	38 (67)	
Antibiotics used			
Yes	267 (99·3)	57 (100)	0·514
No	2 (0·7)	0 (0)	
Steroid used			
Yes	110 (41)	14 (25)	**0·021**
No	159 (59)	43 (75)	
Virus coinfection			
Dual infection			
Yes	69 (26)	6 (11)	**0·014**
No	200 (74)	51 (89)	
Triple infection			
Yes	14 (5)	0(0)	0·078
No	255 (95)	57 (100)	
Multi-infection			
Yes	84 (31)	6 (11)	**0·001**
No	185 (69)	51 (89)	

Significant *P*-values are shown in bold.

## Discussion

This study is the first population-based ARI surveillance conducted in Vietnam that prospectively monitored hospitalized pediatric ARI cases, starting two and a half years prior to the A(H1N1)pdm09 outbreak. Utilizing this unique population-based surveillance, we could accurately measure the incidence rate of hospitalized children <5 years with A(H1N1)pdm09, which was 136 per 100 000 children in central Vietnam, and demonstrated that the A(H1N1)pdm09 did not increase the overall incidence of ARI hospitalization and disease severity compared with seasonal influenza A.

### Influenza A incidence

Our study revealed the incidence of influenza A before, during, and after the 2009 pandemic influenza. The incidence of influenza varies among different countries especially in countries with different geographic location.

#### Pre-pandemic influenza

In Vietnam, the influenza A incidence was 775 and 474 per 100 000 during 2007–2008 and 2008–2009 influenza seasons, respectively. This pre-pandemic incidence was similar to incidence data from Bangladesh which was 670 and 440 per 100 000 in 2008 and 2009, respectively.^21^ In Thailand,^22^ Greece,^23^ and Kenya,^24^ the influenza pneumonia incidence was slightly lower: 236 per 100 000 during 2005–2008 periods, 312 in 2005, and 144 in 2007–2009 periods, respectively. Much lower incidences of influenza hospitalization were observed in the United States^25^ (25·9–41·1 per 100 000 for 2006–2009 periods).

#### 2009 pandemic influenza

During the A(H1N1)pdm09 outbreak, compared with our data, higher incidence rates were reported from neighboring countries such as Singapore^26^ (177 per 100 000) and Thailand^27^ (214 per 100 000), but lower rates were reported from United States^25^ (79·8 per 100 000), Australia^28^ (61 per 100 000), Japan^14^ (40 per 100 000), and Malaysia^29^ (24 per 100 000).

#### Post-pandemic incidence

In our study during the post-pandemic period (2010 April to 2011 March), even though the overall influenza A incidence rate did not increase (452 versus 387 per 100 000), the A(H1N1)pdm09 incidence among children <5 years of age slightly increased from 136 to 194 per 100 000 in the post-pandemic season. Similarly, in England, the incidence of A(H1N1)pdm09 hospitalization increased two times from 2·6 to 5·3 per 100 000 during the post-pandemic periods.^13^ In contrast, in Argentina where the A(H1N1)pdm09 hospitalization rate among children increased 2 times during 2009 pandemic, no pediatric hospitalization due to A(H1N1)pdm09 was identified in the post-pandemic season.^30^ Similar reduction in influenza cases in the post-pandemic period was also reported in the United Sates from 79·8 during the pandemic to 36·5 in the next influenza season.^31^

In this study setting, the influenza A hospitalization incidence for both seasonal and pandemic was highest among 12–23 month age group. This may be due to the waning of the protective effect of maternal transferred antibody to the child and the changing social contact behavior in this age group. Several factors may be responsible for the difference of influenza incidence in different countries. First, influenza surveillance and detection system might be different between during and after the A(H1N1)pdm09 pandemic in some countries. As many countries expanded the surveillance system and switched from rapid antigen test to PCR-based detection which is more sensitive, it is expected to have increased detection rates. Second, each country adopted various management programs to control the outbreak during the pandemic, including school closure, antiviral treatment, and behavior change due to pandemic awareness. They will also have effect on the outcome. Lastly, influenza seasonality is naturally different between temperate and tropical regions. In connection with that, influenza virus survival and transmissibility, host physiology, social behavior, or their combination can contribute to different epidemiological patterns.

### Clinical severity of pandemic influenza

During pandemic influenza epidemic in 2009, there were several reports regarding the disease severity of A(H1N1)pdm09 especially from Mexico^32^, Argentina^6^, and the United States.^5^,^25^ In our study, we found that the A(H1N1)pdm09 cases were older children, came to the hospital earlier after the onset (median 1·6 days), had lower rate of RCP (11% versus 27%) and milder symptoms compared with seasonal influenza A cases.

In Vietnam, the health care for all children under 6 years of age is free of charge and their access to medical facilities in our study site is relatively good. However, to receive free treatment, they need to go to designated commune health center or polyclinic first to receive clinical treatment. If illness is severe, they will be referred to KHGH. On June 11, 2009, the WHO declared globally the A(H1N1)pdm09 pandemic influenza outbreak. In Vietnam, the government informed all medical institution and the public about the situation and introduced guidelines for management. The first case of A(H1N1)pdm09 was detected in Nha Trang on July 30, 2009. At that time, the community was already aware of the situation, so this might led the parents to bring their children to the health facilities earlier. This changing behavior may be one of the reasons for seeing less severe cases among A(H1N1)pdm09 cases compared with seasonal influenza cases in Vietnam. However, as only the severe cases will be admitted to KHGH, this effect should be minimal.

Our finding was supported by similar reports from Canada,^33^ United States,^34^ the Netherlands,^35^ and Australia^36^ which also reported that A(H1N1)pdm09 cases did not increase the hospitalization period and disease severity compared with seasonal influenza cases.

### Less viral coinfection cases among the pH1N1 cases

Epidemiologic patterns of cocirculating respiratory viruses are important because they could affect influenza epidemic due to non-specific host responses to viral infections and different viral–viral interactions. Coinfection with other respiratory viruses, especially RSV, was reported to increase the disease severity of A(H1N1)pdm09.^6^,^37^ In our study, A(H1N1)pdm09 cases had lower viral coinfection rate, and dual infection with A(H1N1)pdm09 and RSV was not observed. This may be the reason why lower rate of wheezing and disease severity was observed among A(H1N1)pdm09 cases in Vietnam.

Other potential contributory factors for disease severity may be secondary bacterial coinfection in A(H1N1)pdm09 cases, socio-demographic factors including access to the hospital, socio-economic status and health-seeking behavior. Underlying risk factors such as malnutrition, vitamin D deficiency, and other underlying clinical conditions might also be responsible. Genetic background may also play a role because a polymorphism at IFITM3 allele (SNP rs 12252-C) has been shown to cause a reduced influenza virus restriction.^38^

### Limitations of the study

We were not able to genotype all influenza A-positive cases, mainly because some clinical samples contained relatively low copy numbers of influenza A virus that restricted viral RNA extraction in good quantity and quality to perform the genotyping assays. However, the number of the samples that we could not perform genotyping was only 28 after the pandemic influenza A; therefore, it is less likely to affect the main outcome of the study. A large portion of samples from 2007 and 2008 (*n* = 91) was not available for genotyping due to lack of samples. The study only covered the hospitalized pediatric influenza cases, so the study could not determine the potential changes among mild influenza cases treated at outpatient department or those who did not come to the study hospital in the region. In conclusion, the pandemic influenza A outbreak in 2009 did not increase the rate of pediatric ARI hospitalization and disease severity in central Vietnam. Public health intervention to promote influenza vaccine usage can reduce the burden of influenza in Vietnam.
